# Health Risks and Changes in Self-Efficacy Following Community Health Screening of Adults with Serious Mental Illnesses

**DOI:** 10.1371/journal.pone.0123552

**Published:** 2015-04-13

**Authors:** Judith A. Cook, Lisa A. Razzano, Margaret A. Swarbrick, Jessica A. Jonikas, Chantelle Yost, Larisa Burke, Pamela J. Steigman, Alberto Santos

**Affiliations:** 1 University of Illinois at Chicago, Department of Psychiatry, Chicago, Illinois, United States of America; 2 Rutgers University and Collaborative Support Programs of New Jersey, Freehold, New Jersey, United States of America; 3 Georgia Regents University, Department of Psychiatry, Augusta, Georgia, United States of America; University of Pennsylvania, UNITED STATES

## Abstract

Physical health screenings were conducted by researchers and peer wellness specialists for adults attending publicly-funded community mental health programs. A total of 457 adults with serious mental illnesses attended health fairs in 4 U.S. states and were screened for 8 common medical co-morbidities and health risk factors. Also assessed were self-reported health competencies, medical conditions, and health service utilization. Compared to non-institutionalized U.S. adults, markedly higher proportions screened positive for obesity (60%), hypertension (32%), diabetes (14%), smoking (44%), nicotine dependence (62%), alcohol abuse (17%), drug abuse (11%), and coronary heart disease (10%). A lower proportion screened positive for hyperlipidemia (7%). Multivariable random regression analysis found significant pre- to post-screening increases in participants’ self-rated abilities for health practices, competence for health maintenance, and health locus of control. Screening identified 82 instances of undiagnosed diabetes, hypertension or hyperlipidemia, and 76 instances where these disorders were treated but uncontrolled. These results are discussed in the context of how this global public health approach holds promise for furthering the goal of integrating health and mental health care.

## Introduction

People with serious mental illnesses experience multiple health disparities [1], higher medical morbidity [2], and lifespans 10–30 years shorter on average than the general population [3]. These disparities exist in countries with universal access to healthcare and in countries with healthcare systems that are highly regarded [4]. Yet we have little detailed knowledge about the prevalence of medical co-morbidities in subgroups of this population, such as those receiving public mental health services in outpatient settings [5–6]. We report on a project conducted collaboratively by a university research center and a peer health and wellness promotion program in which physical health screenings were conducted for clients of community mental health programs located in four U.S. states [7].

Among adults with serious mental illnesses, the most common debilitating physical illnesses are preventable conditions including cardiovascular, respiratory, metabolic, and infectious diseases [8]. Some of these conditions result from the use of psychotropic medications and their related side effects [9–10]. Others are attributed to high fat/low fiber diets [11] with high volume of food intake [12–13]; sedentary lifestyles devoid of exercise and other forms of physical activity [14–15]; lack of screening for early detection [16–17]; and limited access to health care [18–19]. Finally, adults with serious mental illnesses may underutilize medical care due to low levels of health literacy [20–21] and perceived stigma from medical providers [22–23].

One public health strategy for health promotion and prevention involves conducting community health screenings, also known as health fairs [24], which have been implemented globally to address multiple health risks [25–30]. Screening has been used successfully in a variety of settings including schools, workplaces, libraries, places of worship, community organizations, senior centers, and other locations [31–35]. These events help community members who underutilize health care to learn about health risks and available services [36], expose them to health care workers and common medical tests [37], and provide health-related networking opportunities [38]. Previous research has demonstrated that participation in health screening positively affects health beliefs, including feelings of control over one’s health, as well as an increased sense of health responsibility and health self-efficacy [38–39]. Screening also can serve as a “cue to action,” increasing the likelihood that participants will seek treatment or initiate preventive behaviors for identified risks [40–42]. Health screening also enhances participants’ general health knowledge and provides personalized information about health risks [38,43]. In fact, studies show that most individuals attend health screenings to directly access health information relevant to their specific needs [44]. Finally, screening has been used successfully for participants with physical [45], intellectual [46], and learning disabilities [47].

Few studies report on the results of general health screening for people with serious mental illnesses, also known as psychiatric disabilities [48]. A program of annual screening for outpatients with schizophrenia and bipolar disorders in Scotland [49] found that 75% had abnormal results, including overweight or obese Body Mass Index (BMI) in 67%, hypertension in 14%, abnormal urinalysis (e.g., hematuria, proteinuria) in 24%, and abnormal biochemistry (e.g., lipid abnormalities) in 46%. Screening of New York state mental health clinic clients [50] found that 79% were overweight or obese, 27% were hypertensive, and 50% were active tobacco smokers. Finally, cardiometabolic screening of U.S. outpatients at public mental health clinics and group practices [51] found that 27% were overweight and 52% were obese; 51% were hypertensive; and 51% had elevated triglycerides.

The past several decades have witnessed the rapid growth of peer-operated mental health mutual support programs [52] and, increasingly, evidence-based peer-led services [53–54], including health promotion and disease management [20–21,55]. Peers trained as health and wellness specialists now provide health education to people with psychiatric disabilities [56], and offer direct assistance with accessing medical treatment as “peer health navigators”[57]. In some states, such as Georgia, peer services are reimbursable by Medicaid [58]. In the present study, university researchers collaborated with peer health specialists to design and conduct a series of three-day health fairs for people with serious mental disorders. The purpose of the study was to screen for, identify, and document co-occurring physical health conditions, health risk factors, and use of health care. Another goal was to assess potential improvement in health attitudes and self-efficacy following fair participation. We hypothesized: 1) that the point prevalence of all conditions and risks for which we screened would exceed those found in the general U.S. population of non-institutionalized adults; and 2) that health self-efficacy would improve following screening.

## Methods

### Study population and screening procedures

Eligibility criteria included serious mental illness as defined by U.S. federal Public Law 102–321 [59] to include a *DSM-IV-TR*[60] diagnosis of mental illness accompanied by moderate to severe functional impairment; age 18 years or older; status as a client of the participating community mental health program; and ability to provide informed consent. The study used a “controlled intervention” design common in health screening research [30] that targeted specific communities, in this case defined as clients of particular mental health organizations served in programs for people with serious mental illness. The first screening was held at a peer-operated self-help program in New Brunswick, NJ, and attended by members of peer-run centers across the state. The second took place in the gymnasium of a university in Chicago, IL, and was attended by clients of a psychiatric rehabilitation agency located city-wide. The third was held in an Elk’s Club Lodge and a church auditorium, and attended by clients of mental health agencies in Frederick and Rockville, MD (respectively). The fourth occurred at a large community mental health agency located in Smyrna, GA. Participants were recruited by program staff through clinical referral, flyers, announcements at membership meetings, waiting room posters, and word-of-mouth. While clinicians helped with recruitment, participation was strictly voluntary, and clinicians were not informed about whether their clients attended the health fairs. The University of Illinois at Chicago (UIC) Institutional Review Board (IRB) and the Sheppard Pratt Health System IRB approved this study including its informed consent procedures. Written informed consent was obtained from participants on the day of the screening by trained researchers, using procedures approved by the UIC and Sheppard Pratt IRBs. Capacity to provide informed consent was assessed by trained researchers with questions that assessed the person’s understanding of the purpose of the research and foreseeable risks and anticipated benefits of study participation. No surrogate consent procedure was used. Only 2% (7 of 464) of those attending the screenings refused participation.

Following informed consent, participants received a number-coded “health passport” in which their test results were recorded, along with a wrist band containing a corresponding number. This allowed individuals to be matched with test results without collecting their names. Next, participants completed a pre-test interview, as described below. They then visited each of the testing stations, at which screening staff performed tests and recorded results in the health passport, and on a coded sheet maintained at each station.

After testing, participants met with trained peer health and wellness specialists who reviewed their test results, provided health education, and offered peer support. Following this meeting, participants completed a post-test interview while the screening results from their health passports were being entered into a data base. These results were later verified against those maintained at testing stations. At check-out, health passports were returned to participants by matching the numeric code on their wrist bands. They received gift cards from local stores and gift bags containing wellness products to thank them for their time.

Given prior studies showing that health fair participants increased their knowledge about health risks and adopted new health behaviors, fairs were designed to promote these processes. Screening activities allowed for dialogue between staff and participants at each station, including immediate feedback about test results, education regarding their meaning, chances to ask questions, and one-page handouts for later reference. At the final station, they met with peer health and wellness specialists who reviewed test results in their entirety, answered questions, and engaged in dialogues about potential strategies for addressing health risks. Peer staff focused on what participants could do in the immediate future, using a handout containing potential actions for different health risks. Finally, participants received a list of sources of free health care and further testing in their local communities, along with encouragement to follow up.

Screening staff included medical students, psychiatry residents, researchers, and peer specialists, each of whom completed a minimum of 6 hours of education for each test administered, including observation and corrective feedback. All serologic testing was conducted by licensed nurses or other medically trained staff. Throughout the screening process and afterwards, support and reassurance was available from the peer health and wellness specialists. To further increase participants’ comfort levels, additional peers from each local agency were recruited, trained, and paid to work as health fair greeters and logistics coordinators, answering questions and guiding participants between screening stations.

### Measures

#### Attitudinal measures

A standardized research protocol was administered at pre- and post-screening by trained interviewers to collect demographic information, medical history, health attitudes, and use of health care services. Ability to engage in health maintenance practices was assessed by the Responsible Health Practices subscale of the Self-Rated Abilities for Health Practices Scale [61]. Participants responded to 7 items such as “I recognize what symptoms should be reported to a doctor or nurse” using a 5-point Likert scale ranging from 0 (not at all) to 4 (completely) and referring to the present time. Inter-item reliability for this measure was good with alpha = .82 for pretest and .84 for post-test. Competence in health self-management was assessed using the Perceived Competence for Health Scale [62], adapted from a scale measuring self-management of diabetes. Participants responded to 4 items such as “I am capable of handling my health needs now” using a 7-point Likert scale ranging from 1 (not at all) to 7 (very true) and referring to the present time. Inter-item reliability for this measure was good with alphas of .82 for pre-test and .86 for post-test. The Multidimensional Health Locus of Control Scale (HLOC)[63] is comprised of 3 subscales that measure the degree to which individuals feel they can affect their health and health-related behaviors. The Internal subscale assesses respondents’ degree of perceived control over their own health and consists of 6 items such as “I am directly responsible for my own health” using a 6-point Likert scale ranging from 1 (strongly disagree) to 6 (strongly agree) and referring to the present time. The Powerful Others subscale assesses the degree to which respondents believe their health is controlled by other individuals and includes 6 items such as “Health professionals control my health” rated using the same 6-point scale and referring to the present. The Chance subscale assesses the degree to which respondents believe their health is due to chance factors and includes 6 items such as “Most things that affect my health happen to me accidentally” using the same 6-point scale and referring to the present time. Pre- and post-test alphas for these subscales ranged from .60 to .74.

Prevalence of physical health conditions was assessed using items from the National Health Interview Survey (NHIS)[64] and the National Health and Nutrition Examination Survey (NHANES)[65]. Respondents were asked whether they had ever been told by a doctor or other health professional that they had a series of medical conditions and, if so, whether they still had the condition and were in treatment for it.

#### Health risk assessments

Measures of health risk included body mass index (BMI) based on height and weight; blood pressure; blood glucose; and non-fasting lipid profile including total cholesterol, high-density lipoprotein (HDL), low-density lipoprotein (LDL) and triglycerides. Glucose and lipid profiles were obtained using the Cholestech LDX system (Inverness Medical, Hayward, CA). Risk for alcohol abuse/dependence was assessed using the Alcohol Use Disorders Identification Test—Consumption (AUDIT-C)[66]; risk for substance abuse/dependence using the Drug Abuse Screening Test (DAST)[67]; and nicotine dependence using the Fagerstrom Test for Nicotine Dependence [68]. Estimated 10-year risk of coronary heart disease was determined using the Framingham risk model [69] and calculated at the Cybermed website (Cybermed, 2000) based on the participant’s age, sex, systolic blood pressure, total and HDL cholesterol, diabetes mellitus, and current smoking status. Using the tertiles of their Framingham risk score, participants were classified as being at “high,” “intermediate,” or “low” risk for heart disease.

#### Background characteristics

Participants self-reported demographic and other personal characteristics during the interviews including age, race/ethnicity, education, psychiatric diagnosis, and health insurance status.

### Statistical analysis

Data were downloaded into SPSS [70] and descriptive statistics (means, medians) were calculated to create prevalence scores. Risk for each condition was defined according to standardized indicators and cut-off values contained in evidence-based practice guidelines from the National Institutes of Health (NIH) and Centers for Disease Control and Prevention (CDC). Random regression analysis using SuperMix [71] tested for pre- to post-screening changes in participants’ perceived health efficacy and competency, controlling for person-level factors of sex, race, age, and education, as well as study site.

## Results

### Background characteristics and representativeness

Table 1 presents participants’ demographic and clinical characteristics. Comparison to adult populations at each agency revealed no significant differences by sex, race, Hispanic/Latino ethnicity, education, age, diagnosis, and health insurance status with two exceptions. In NJ, a smaller proportion of males were screened (46%) than in the agency population (59%) (Z = -2.1, p<.05). In GA, more screening participants reported schizophrenia (40%) than the agency population (25%) (Z = 3.3, p<.001). Otherwise, screening participants were representative of each population targeted.

**Table 1:** Characteristics of adults with serious mental illnesses screened for common health conditions by U.S. state and total (N = 457)^1^.

	Total		New Jersey		Illinois		Maryland		Georgia	
	N = 457		n = 121		n = 122		n = 106		n = 108	
	N	%	N	%	N	%	N	%	N	%
Male	236	51.3	56	46.3	81	66.1	60	55.8	39	36.1
Race										
White/Caucasian	221	48.8	62	51.2	34	28.1	67	65.0	58	53.7
Black/African American	175	38.6	41	33.9	69	57.0	25	24.3	40	37.0
Asian/Pacific Islander	7	1.5	2	1.7	2	1.7	3	2.9	1	0.9
American Indian/Alaskan Native	2	0.4	0	0.0	2	1.7	0	0.0	0	0.0
Multi-Racial	17	3.8	6	5.0	3	2.5	4	3.9	4	3.7
Other	30	6.6	10	8.3	11	9.1	4	3.9	5	4.6
Hispanic/Latino Ethnicity	32	7.1	13	10.8	13	10.8	5	4.8	1	0.9
Education										
< High School	89	20.0	16	13.8	38	32.2	18	17.1	17	16.2
High School/GED	138	31.1	30	25.9	34	28.8	30	28.6	44	41.9
Some College/Advanced Degree	217	48.9	70	60.3	46	39.0	57	54.3	44	41.9
Mean (SD) age, years	46.5(12.1)		49.1(13.4)		47.1(11.1)		44.6(12.3)		44.7(11.0)	
Health Insurance Type										
Medicaid	130	29.2	37	31.6	42	35.6	34	32.4	17	16.2
Medicare	82	18.4	28	23.9	18	15.3	20	19.0	16	15.2
Dual	137	30.7	31	25.6	43	36.4	42	40.0	21	20.0
Private	43	9.7	21	17.9	6	5.1	14	13.3	2	1.9
Veteran’s	11	2.5	4	3.4	2	1.7	1	1.0	4	3.8
Other	22	5.2	7	6.0	9	7.6	5	4.8	2	1.9
None	62	13.9	6	5.1	7	5.9	3	2.9	46	43.8
DSM-IV Diagnosis										
Schizophrenia	179	40.6	26	21.8	63	53.8	48	48.5	42	39.6
Bipolar Disorder	100	22.7	31	26.1	17	14.5	24	24.2	28	26.4
Depression	106	24.0	34	28.6	31	26.5	19	19.2	22	20.8
Anxiety Disorder	19	4.3	4	3.4	2	1.7	4	4.0	9	8.5
Personality Disorder	4	0.9	2	1.7	0	0.0	2	2.0	0	0.0
Other	33	7.5	22	18.5	4	3.4	2	2.0	5	4.7
Taking Psychiatric Medicine	294	88.8	—	—^2^	108	89.3	90	86.5	96	90.6

^1^ Variations in N due to missing data

^2^ Question not asked at this site

### Prevalence in the study and U.S. general population

The large majority of health fair participants (82%, n = 370) were either overweight (BMI = 25.0–29.9) or obese (BMI>29.9), at 22% (n = 100) and 60% (n = 270) respectively (Table 2). Total cholesterol was elevated in 24% (n = 103), with 17% (n = 75) classified as “slightly high” (201–239 mg/dL) and 7% (n = 28) as “high” (240+ mg/dL). Hemoglobin A1c (HbA1c) values met criteria for pre-diabetes (HbA1c = 5.7–6.4%) among 25% (n = 108) and for diabetes (HbA1c≥6.5%) among 14% (n = 62). Blood pressure readings indicated that 37% (n = 169) were pre-hypertensive (BP = 120–139/80–89), and 32% (n = 145) had hypertension (BP≥140/90).

**Table 2:** Results of health risk assessments and comparison with U.S. population (N = 457)^1^.

Health/Risk Assessment	Screened Population		% At Risk in U.S. Population	% At Risk of those Screened
	%	N		
Obesity/Body Mass Index				
18.5 or less—Underweight	1	7		
18.6–24.9—Normal	17	75		
25.0–29.9—Overweight	22	100		
30+—Obese	60	270	36^2^	60
Hyperlipidemia/Total Cholesterol				
<200 mg/dL—Healthy	76	330		
201–239 mg/dL—Slightly High	17	75		
240+ mg/dL—High	7	28	13^3^	7
Diabetes/Hemoglobin A1c				
4–5.6%—Balanced	61	264		
5.7–6.4%—Prediabetes	25	108		
6.5%+—Diabetes	14	62	2^4^	14
Hypertension/Blood Pressure				
<120/80—Normal	31	139		
120–139/80–89—Pre-Hypertensive	37	169		
140+/90+—Hypertensive	32	145	29^5^	32
Proportion Smoking	44	200	19^6^	44
Nicotine Dependence/Fagerstrom				
0–3—Very Low/Low Dependence	38	75	43^6^	
4–10—Medium/High Dependence	62	121	57^6^	62
Alcohol Abuse/Audit-C				
No Risk	83	371		
At Risk	17	75	7^7^	17
Drug Abuse/DAST				
No Risk	79	351		
Low Risk	10	46		
Intermediate/Substantial/Severe Risk	11	48	3^7^	11
Coronary Heart Disease/Framingham				
≤10%—Low	78	344		
11–19%—Medium	12	54		
≥20%—High	10	44	3^8^	10

^1^ Variation in sample size due to missing values (i.e., refusals and nonreactive tests)

^2^ National Health and Nutrition Examination Survey (Flegal et al., 2012)

^3^ National Health and Nutrition Examination Survey 2009–2010 (Carroll et al., 2012)

^4^ National Health and Nutrition Examination Survey 2007–2009 (CDC, 2011)

^5^ National Health and Nutrition Examination Survey 2009–2010 (Yoon et al., 2012)

^6^ National Survey on Drug Use and Health 2006 (SAMHSA, 2008)

^7^ National Survey on Drug Use and Health 2010 (SAMHSA, 2010)

^8^ National Health and Nutrition Examination Survey III (Ford et al., 2004)

A sizable minority (44%, n = 200) reported that they currently smoked tobacco. Of these, 62% (n = 121) met criteria for medium to very high nicotine dependence. Seventeen percent (n = 75) of those screened were at risk for alcohol abuse. A smaller but noteworthy proportion (11%, n = 48) were at risk for drug abuse. The prevalence of an estimated 10-year coronary heart disease risk of ≥20%, considered “high,” was 10% (n = 44), “intermediate” risk of >10% to <20% was 12% (n = 54), and “low” risk of ≤10% was 78% (n = 344).

Compared to prevalence reported for the non-institutionalized adult U.S. population, proportions were higher for all but one of the conditions screened. Sixty percent of outpatients with mental illnesses were obese compared to 36% in the general population [72]. Fourteen percent had HbA1c values indicating diabetes compared to 2% of the general population [73]. Thirty-two percent had blood pressure indicating hypertension compared to 29% in the general population [74]. Forty-four percent were current smokers compared to 19% in the general population [75], and nicotine dependence was medium to high in 62% compared to 57% in the general population [76]. Seventeen percent screened positive for alcohol abuse based on their current consumption compared to 7% prevalence in the general population [77]. Eleven percent screened positive for substance abuse compared to 3% prevalence in the general population [77]. Finally, 10% of participants screened high for coronary heart disease risk compared to their age and sex cohorts, while 3% of the general population was found to be at risk [78].

The one exception to this pattern was total cholesterol, with hyperlipidemia being less prevalent among participants with serious mental illnesses (7%) compared to the general U.S. population (13%)[79].

### Undiagnosed, untreated, and unsuccessfully treated conditions

Using items from the NHANES III [80], we determined the proportion of participants ever diagnosed with three of the medical conditions for which they were screened, as well as the proportion currently being treated. Among the 62 participants with HbA1c levels indicating diabetes (≥6.5), 24% (n = 15) said they had not received a diagnosis of diabetes. Among the 96 participants reporting a diabetes diagnosis, 10% (n = 10) did not report current treatment. Among the 86 reporting current treatment, HbA1c levels indicated diabetes in 50% (n = 43). Thus, the screening identified 15 individuals with undiagnosed diabetes, and 43 with treated but uncontrolled diabetes.

Among the 145 who screened positive for high blood pressure (BP≥140/90), 43% (n = 60) said they had not received a diagnosis of hypertension. Among the 197 participants reporting a diagnosis of hypertension, 24% (n = 47) did not report current treatment. Among the 150 individuals reporting current treatment, high blood pressure was detected among 41% (n = 61). Thus, 60 individuals had undiagnosed hypertension, and 61 had treated but uncontrolled hypertension.

Among the 28 participants who screened positive for high cholesterol (≥240 mg/dL), 25% (n = 7) said that they had not been diagnosed with hyperlipidemia. Among the 198 participants reporting a diagnosis of high cholesterol, 32% (n = 64) did not report receiving current treatment. Among those reporting current treatment and with a reactive test, 9% (n = 12) had high cholesterol levels. Thus, screening identified 7 individuals with undiagnosed high cholesterol, and 12 with treated but uncontrolled high cholesterol.

### Changes in health self-efficacy following screening

We tested for changes in health attitudes from pre- to post-screening. Multivariable random regression analysis controlling for age, sex, race, education, and study site revealed small but statistically significant increases in self-rated abilities for health practices, and in perceived competence for health maintenance (Table 3). Significant increases also were observed in two of the three health locus of control subscales, namely internal control, and powerful others. There were no significant changes in participants’ perceptions of their health being controlled by chance factors.

**Table 3:** Random regression analysis of changes in health self-efficacy among adults with serious mental illnesses pre- and post-health screening, controlling for sex, age, race, education, and study site (N = 457).

Health/Self-Efficacy Measure	Pre	Post	Estimate^1^ (SE)	Z Score	P Value
	*x̄*	*x̄*			
Self-Rated Abilities for Health Practices^2^	14.6	15.4	0.71 (0.22)	3.26	.001
Perceived Competence for Health Maintenance^3^	21.2	22.5	1.27 (0.25)	5.04	<.001
Multidimensional Health Locus of Control Factors					
Internal Control^4^	27.5	28.0	0.56 (0.24)	2.29	.02
Powerful Others^5^	23.0	24.7	1.73 (0.34)	5.11	<.001
Chance^6^	19.2	19.4	0.28 (0.29)	0.95	.34

^1^ Unstandardized random regression estimate (SuperMix) where sign indicates direction of effect.

^2^ Higher score indicates better perceived ability to engage in health practices, min/max = 0–28

^3^ Higher scores indicates higher perceived competence for health maintenance, min/max = 4–28

^4^ Higher score indicates greater internal control over one’s health, min/max = 6–36

^5^ Higher score indicates greater control of powerful others over one’s health, min/max = 6–36

^6^ Higher score indicates greater role of chance in one’s health, min/max = 6–36

## Discussion

To our knowledge, this is the first study to screen for a comprehensive set of health risks among people serious mental illness receiving outpatient treatment in multiple regions of the U.S., providing same-day results to participants along with health education and peer support. Ours is also the first to involve peer health and wellness specialists in the design and implementation of screening activities, and the first to demonstrate changes in health self-efficacy among participants from pre-to post-screening. Compared to prevalence reported for the non-institutionalized U.S. adult population, we found that higher proportions of participants screened positive for obesity, diabetes, hypertension, smoking and nicotine dependence, alcohol abuse, drug abuse, and coronary heart disease. Only in the case of hyperlipidemia was the proportion lower in the participant group than in the general population. Our results are also highly similar to those found in three prior screening studies with this population [48–50].

As hypothesized, positive changes occurred in self-perceived health efficacy following participation in health screening. At post-test, participants gave higher ratings to their degree of competence in performing health practices and to their ability to engage in health maintenance activities. In addition, they reported an enhanced degree of internal control over their own health. However, they also showed an increase in the degree to which they felt that “powerful others” exerted control over their health. The latter finding may be due to their recent interactions with our screening staff who they may have perceived as “powerful” health experts. It may also be due to feelings of powerlessness and low self-efficacy in general that have been found in prior studies of this population [81–83].

Given that participants spent a modest amount of time engaging in health fair activities, totaling 60–90 minutes on average, even small increases in positive health attitudes and self-rated competencies are noteworthy. The changes we observed suggest that health fair follow-up activities might be beneficial in enhancing any gains that participants experienced [43,84]. For example, a study of individuals attending the Indiana Black and Minority Health Fair [85] found that those who received health counseling sessions afterwards were more likely than controls to report improvement in general health status and healthy behaviors at 15-month follow-up.

Health screening also was successful at detecting serious medical problems of which participants were either unaware or for which they were receiving treatment but did not demonstrate values in the expected normal ranges. Screening identified 82 instances of undiagnosed diabetes, hypertension or hyperlipidemia, and 76 instances where these disorders were treated but uncontrolled. This noteworthy level of unmet needs suggest that many would benefit from enhanced coordination with medical providers to address health risks.

One advantage of this approach to screening is that participants with newly identified health risks and enhanced health self-efficacy can immediately be provided with information about the nature and treatment of each condition, along with peer support for decision-making about next steps. Peer health and wellness specialists assessed participants’ readiness for change in order to empower and encourage people, no matter what their planned course of action or inaction. For those expressing a desire to act, peer staff emphasized simple lifestyle alterations that participants could make in the near future, using a handout that cross-walked different health risks with potential life changes. For example, participants who expressed readiness to address high cholesterol were encouraged to add fiber to their diets, eat frequent small portions instead of three big meals, reduce or eliminate sweets and alcohol, and record what they ate in a food diary. Those with high nicotine dependence learned about new medications and psychosocial interventions for smoking cessation. Those not ready to quit were informed about the health advantages of reducing their smoking, even without complete cessation.

Given documented health disparities in this population, our finding of lower prevalence for one chronic condition among participants than in the general population is noteworthy. This was hyperlipidemia, where 7% of our participants screened positive for high serum total cholesterol compared to 14% in the NHANES [79]. This may be due to the absence of a non-fasting test that may have under-estimated lipid abnormalities. Another possibility is that hyperlipidemia is more easily managed in concert with serious mental illness than are other co-occurring medical conditions. Our participants are among the one-in-five Americans managing multiple chronic conditions [86], and research suggests that certain clusters of co-morbid illnesses are more appropriately treated (e.g., depression and hyperlipidemia)[87] than others (e.g., psychosis and arthritis)[88]. Whatever the reason, this finding underscores the potential for those in the mental health field to partner with peer health specialists and primary care providers in efforts to screen for and successfully control co-morbid medical risks in this vulnerable population.

A number of caveats apply to our study findings. First, study participants were not nationally representative of adults with serious mental illness since they were recruited from mental health programs in only four U.S. states. Second, without a control group, we are unable to attribute the pre-post changes in health attitudes we observed to health fair participation itself. Third, we were unable to conduct follow-up testing to confirm our screening results and, thus, we do not know the proportion of “false negative” or “false positive” results that our testing yielded. Fourth the self-report nature of the NHANES questions introduces potential biases for operationalizing prevalence and treatment statuses given that respondents may not recall or may incorrectly report certain medical conditions and services. Fifth, administering the post-test assessment immediately after the health fair did not allow participants time for changes in health behaviors that might have been reflected in the attitudinal measures. Finally, the mental health agencies that participated may be more “health-conscious” than typical community mental health programs and, thus, their clients may be more empowered to take care of their health and healthier as a result.

Anecdotal observations shared with us by agency staff indicated that, after the screenings, many participants brought their health passports to meetings with their mental health clinicians and primary care providers to review test results and discuss future courses of action. Both during and after the screenings, participants expressed positive reactions to their contacts with peer health and wellness specialists who staffed the events. For many, this was their first exposure to peers in this role and they inquired about how to receive similar training. In a few cases, the local agency peers we hired and trained as greeters and logistics coordinators went on to complete their state’s peer specialist certification or other health-related training. Another project outcome was the creation of a health screening manual for people in community mental health programs called “Promoting Wellness for People in Mental Health Recovery” that is available for free download at http://www.cmhsrp.uic.edu/health/designing_health_screening.asp.

In conclusion, our results suggest that collaborative health risk screening involving peers can help to answer epidemiologic questions, provide targeted health education, and empower participants to better manage their medical needs. Given the global use of community screening as a tool for addressing health disparities, the fields of psychiatry and psychology, medicine, nursing, social work, occupational therapy, and public health have much to offer these efforts. This might include helping to organize screening events with community partners; encouraging the involvement of medical students, residents and interns; staffing health fair stations; and supporting patients’ participation in screening activities. Through this public health approach, the integration of health and mental health care can be both practiced and promoted to reduce the high levels of morbidity and mortality that impede mental health recovery.
